# 

**Published:** 1873-01

**Authors:** 


### INDEX.

Annual Meeting of the Ohio State Society . . 50
Alkalies . . . . . . . . . 56
Arkansas Whetstone . ' . . . . . 78
A Trial of Fifty Years . . . . . . . 97
Annual Address before the Mississippi Valley Dental Association . . . . . . . . . 155
Address before the Indiana State Dental Association 163
A Diseased Mouth a Source of General Disease . 178
New Dental Journal and a Dental College in Spain 179
Address at the Commencement of the Ohio Dental College . . . . . . . . . 200
Acknowledgment . . . . . . . 227
Artificial Dentistry . . . . . . . 254
Abrasion—A Case . . . . . . . 258
Address . . . . . . . . . 280
Alveolar Abscess . . . . . . . 294
Academy of Natural Sciences . . . . . 303
American Dental Society . . . . . . 309
A New Thing—Nickel . . . . . . 315
Artificial Crowns . . . . . . . 326
A Shanghai Dentist . . . . . . . 355
A Dentist Fined for Giving Chloroform . . . 394
American Dental Association . . . . . 395
American Dental Convention . . . . . 402
Advice to Young Dentists . . . . . . 415
A Scrap of History . . . . . . . 433

American Dental Society of Europe . . . 440
A New File Carrier . . . . . . . 446
Address—Introductory to the Course of Lectures at the Ohio Dental College . . . . . . . 447
A History of American Dentistry and Dental Surgery 486
Artificial Dentistry . . . . . . . . 491
Address . . . . . . . . . 511
Board of Examiners . . . . . . . 96
Brain Stimulants . . . . . . . 383
Blood Letting . . . . . . . . 389
Bromide of Potassium and the Diseases of Dentition 391
Brooklyn Dental Society . . . . . 484
Corrections . . . . . . . . . 51
Criticisms . . . . . . . . . 76
College Association . . . . . . . 97
Cancrum Oris . . . . . . . . 142
Chemistry of the Mouth . . . . . . 202
Care of the Teeth . . . . . . . 220
California State Dental Association . . . 226, 299
Cincinnati Dental Association . . . . 331, 353
Correspondence . . . . . . . 377
Causes of Spontaneous Ignition . . . . 392
Characteristics of English Medical Debates . . 393
Chemical Affinity . . . . . . . 417
Cases and Method of Skin Grafting . . . . 430
Causes of Disease . . . . . . . 237
Close of the Volume . . . . . . . 530
Discussions at the 7th Annual Meeting of the Ohio State Dental Society . . . . . . 87, 118
Dental Jottings . . . . . . . . 100
Discussions before the Mississippi Valley Dental Association . . . . . . . . . 164
Discussions of the Ohio State Dental Society . . 169
Discussions of the Mississippi Valley Dental Society 211
Dental Reunion . . . . . . . . 215
Diagnosis . . . . . . . . . 223
Discussion of Offensive Breath . . . . . 228

Diseased Pulp—Mississippi Valley Dental Association . . . 251
Diagnosis . . . . . . . . . . 265
Dental College . . . . . . . . . . 271
Don't Anæsthetize a Female without a Witness . . . 272, 306
Dr. Joseph Taylor . . . . . . . . 291
Dental Convention . . . . . . . . 307
Dental Meetings . . . . . . . . 311
Dental Miscellanies . . . . . . . . 328
Dentistry—Its History, Present Status, Claims and Relations . . . . . . . . . 361
Dental Therapeutics . . . . . . . . 366
Dental Literature of the Day . . . . . . 370
Dental Fees . . . . . . . . . 375
Death from Inhalation of Chloroform . . . . 421
Dr. Chas. T. Jackson . . . . . . . 428
Dental Education . . . . . . . . 444
Dental Association . . . . . . . . 446
Dental Hygiene . . . . . . . . 454
Dental Education . . . . . . . . 458
Died . . . . . . . . . . . 409
Ethics . . . . . . . . . . 52
Editorial 50, 94, 137, 178, 223, 265, 309, 355, 395, 442, 486
Exostosis . . . . . . . . . . 278
Eastern Ohio Dental Association . . . . . 358
Effects of Oxygen and Carbon on Plants . . . . 429
Eleventh Annual Meeting of the Connecticut Valley Dental Association . . . . . . . . 445
Eighth Annual Meeting of the Ohio State Dental Society 489
Education—Treatment of Tooth-pulp . . . . 503
Filling Teeth . . . . . . . . . 198
False Teeth . . . . . . . . . 395
Fat and Lean . . . . . . . . . 432
Fifteenth Annual Session of the Indiana State Dental Association . . . . . . . . . 480
Gutta Percha . . . . . . . . 23, 94
General Causes of Disease . . . . . . 237
General Culture for the Dentist . . . . . 195

Hymenial . . . . . . . . . 226
Horace Wells . . . . . . . . 480
Handy Method for Hypodermy . . . . 431
Hemorrhage Arrested . . . . . . . 420
Health of the Dentist . . . . . . 528
Illinois and Iowa State Dental Association . . 228
Illinois State Dental Association, 1873 . . . 410
Local Anæsthesia . . . . . . . 141
Longevity of the Professions . . . . . 142
Lime Salts Necessary to Children . . . . 185
Lawrence's File Holder . . . . . . 226
Lessons in Physical Diagnosis . . . . . 228
Large Doses of Chloral . . . . . . 304
Last Words about the Epizooty . . . . 428
Legal Intelligence . . . . . . . 384
Mechanical Dentistry . . . . . 14, 320
Mississippi Valley Dental Association 95, 140, 434, 487
Methods and Materials to be used in Filling Teeth . 205
Meetings of the Cincinnati Dental Association . . 228
Mercurius Vivus . . . . . . . 40, 256
Microscopic Objective . . . . . . 312
Mad River Dental Society . . . . . 404, 488
Michigan State Dental Society . . . . . 398
Medication Made Easy . . . . . . 400
Microscope . . . . . . . . 357
Mad River Dental Society . . . . . . 523
New Treatment for Exposed Pulps . . . . 138
Notice . . . . . . . . . . 143
Nitrous Oxide . . . . . . . . 144
New Jersey State Dental Society . . . . 308
New Orleans Dental Association . . . . 483
Northern Ohio Dental Society . . . . . 341
Ohio State Dental Board . . . . . . 50
Ohio Dental College—Spring Course . . . 64
Obituary . . . . . . . . 97, 98, 141
Offensive Breath . . . . . . . . 104

One-Eighth Objective . . . . . . . 186
Osteophytes . . . . . . . . . 210
Ohio Dental College . . . . . . 225, 311
Operating Chairs . . . . . . . 260
Oregon State Dental Society . . . . . . 247
Our Weights . . . . . . . . . 431
Oregon State Dental Society . . . . . . 349
Personal . . . . . . . 139, 140, 271
Professional Literature . . . . . . 179
Phosphates . . . . . . . . . . 236
Placebo in Dental Practice . . . . . . 297
Phenomena of the Brain . . . . . . . 485
"Professional Ethics" . . . . . . . . 499
Phosphatic Food in Debility . . . . . . 424
Phosphorus in Neuralgia . . . . . . . 426
Rubber Dam—its Uses and Applications . . . . 9
Remarks on Methylene . . . . . . . 457
Raising Giants . . . . . . . . . 427
Rubber—Rubber—Rubber . . . . . . 401
Rose Pearl . . . . . . . . 356, 403
Repointing and Manufacturing Instruments . . . 21
Radical Observations About Teeth . . . . . 462
Six Year Molars . - . . . . . . 19, 72
Semi-annual Re-union of the 7th and 8th District Dental Society of New York . . . . . . 79
Southworth's Operating Stool . . . . . . 64
Stricker's Manual of Histology . . . . . 286
Southern Dental Society . . . . . . . 305
Seventh Annual Meeting of the Ohio State Dental Society . . . . . . . . . . 468
Syncope . . . . . . . . . . 405
Saliva as a Cure for Rheumatism . . . . . 429
Sanitary Value of Light . . . . . . . 387
The Best Means of Preserving the Eye-sight of Dental Practitioners . . . . . . . . 33, 37
ransactions of the American Dental Association . 51
he Rubber Contest . . . . . . . . 54

Treatment of Exposed Pulps . . . . . 465
The Practice of Dental Medicine . . . . 467
The Big Telescope . . . . . . . 484
The Adminisration of Perchloride of Iron . . 485
Tennessee Dental Association . . . . . 513
Transactions of the Iowa State Dental Society . . 531
Transactions of the California State Dental Society 531
Transactions of the Illinois State Dental Society . 532
The Table of Societies . . . . . . 532
Vital Force . . . . . . . . . 28
Victories . . . . . . . . 65

